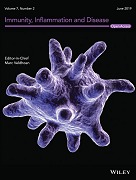# Issue Information

**DOI:** 10.1002/iid3.228

**Published:** 2019-04-26

## Abstract